# Data on medication adherence in adults with neurological disorders: The NeuroGerAd study

**DOI:** 10.1038/s41597-022-01847-9

**Published:** 2022-11-30

**Authors:** Tino Prell, Aline Schönenberg, Sarah Mendorf, Hannah M. Mühlhammer, Julian Grosskreutz, Ulrike Teschner

**Affiliations:** 1 Department of Neurology, Jena University Hospital, Jena, Germany; 2 Department of Geriatrics, Halle University Hospital, Halle, Germany

**Keywords:** Outcomes research, Neurological disorders, Health services

## Abstract

Nonadherence to medication is a common issue that goes along with increased morbidity and mortality and immense health care costs. To improve medication adherence and outcome in ill people, their reasons of not taking their prescribed medication must be known. Here a dataset is presented based on the longitudinal observational NeuroGerAd study in adults with neurological disorders (N = 910). The dataset contains demographic background variables as well as measures of adherence, medication changes after hospital discharge, comprehensive geriatric assessments, personality, patient-physician relationship, and health-related quality of life. As such, the dataset offers unique opportunities to enable a plethora of analyses on personal, social, and institutional factors influencing medication adherence.

## Abstract (summary)

**Table:** 

Measurement(s)	adherence
Technology Type(s)	questionnaires
Factor Type(s)	personal factors
Sample Characteristic - Organism	Homo sapiens
Sample Characteristic - Environment	inpatient and outpatient setting
Sample Characteristic - Location	Germany

## Background & Summary

Adherence is described as the extent to which patients are able or willing to follow agreed recommendations with the medical staff. This includes recommendations on medication, diet, and/or lifestyle changes^1,2^. Adherence plays a particularly important role in chronic illnesses because medication is necessary to be taken continuously as recommended. Its relevance increases for people in older age, as they often have complex therapy regimens due to different diseases^3^. In addition, the relevance of this age group is increasing, as the number of people in older age is rising due to demographic changes^4^. However, many people cannot or do not want to take medications as prescribed^2^. This medication nonadherence leads to adverse drug events, increased length of stay and hospitals readmissions, lower quality of life (QoL), higher costs, and general poorer health outcomes^1,5–7^. Causes for nonadherence are manifold^8^. Furthermore, causes and predictors of nonadherence have been analyzed more frequently for internal diseases such as chronic obstructive pulmonary disease, bronchial asthma, arterial hypertension, etc., and less information are available for neurological diseases in elderly patients^9^.

This paper presents a new dataset that provides unique opportunities to investigate adherence in elderly people with neurological disorders derived from the NeuroGerAd study^10^. The study included a comprehensive geriatric assessment at baseline during hospital stay and two follow-up telephone interviews at 1 and 12 months after hospital discharge. The comprehensive clinical characterization at baseline allowed the determination of patterns and mechanisms of nonadherence. Two follow-up interviews were performed to explore prevalence and reasons of medication changes in the year after hospital discharge. The dataset can be reused for several health-service-research topics, e.g., patterns of depression, mobility, and nonadherence in elderly hospitalized people, or gap between inpatient and outpatient care in Germany.

## Methods

In this observational longitudinal study, data were collected from people who were treated as inpatient at the Department of Neurology, Jena University Hospital, Jena, Germany between February 2019 and March 2020. Procedures included a comprehensive baseline assessment during hospital stay and 2 follow-up interviews at 1 and 12 months after hospital discharge. Baseline assessments included demographical data, clinical data, self-report adherence, prescribed medication, mobility, depression, cognition, health care utilization, communication, personality, and health-related QoL. Follow-up interviews asked for changes of medication after discharge, reasons thereof, specific kind of change, and health-related QoL.

### Setting and participants

This observational and cohort study was registered in the German Clinical Trials Register DRKS00016774 (registered February 19, 2019), and the study protocol was published prior^10^. The study was approved by the local ethics committee (approval number 5290-10/17) of Jena University Hospital. All patients provided written informed consent. Hospitalized elderly patients with neurological disorders received baseline assessment between February 19, 2019 and March 13, 2020; the first telephone follow-up took place between March 19, 2019 and April 13, 2020; and the second telephone follow-up took place between February 19, 2020 and March 14, 2020.

A total of 2,021 patients aged 60 years and older were admitted to the Department of Neurology during the baseline data collection phase; however, 113 were missed for timely reasons, thus, assessments were impossible before their discharge. Of the remaining 1,908 patients that were screened for initial eligibility, 997 were excluded because of a score of <19 points in The Montreal Cognitive Assessment (MoCa) (n = 623) or delirium (n = 27), and because they declined to participate (n = 44), or were hindered to participate due to other medical reasons, such as inability to speak, unconsciousness, or severe dyspnea (n = 259). With the onset of corona virus disease-2019 (COVID-19) pandemic and decreased in the number of patients hospitalized for non-COVID-19-related reasons in January 2021, 136 patients aged between 55 and 60 were included when multimorbidity was present. This was done to gain higher sample size. In total, 995 patients were deemed eligible, of whom 910 patients completed the baseline study. In the first follow-up, 727 (79.9%) participants were interviewed by telephone (8 declined to participate and 175 were unreachable). In the second follow-up after 12 months from 910 participants, 673 (74%) participants were interviewed (27 declined and 210 were unreachable).

Outcome: The primary outcome was nonadherence according to the Stendal Adherence with Medication Score (SAMS). This study aimed to determine the predictors of nonadherence in patients with neurological disorders taking personal, environmental, and procedural factors into consideration.

### Assessments

Several variables were obtained via medical records, self-report, and face-to-face investigation by trained study staff. Questionnaires and assessments are detailed in the Table 1. The full survey form can be found in the dataset repository. Cognition testing was done using the MoCa after explaining the study and obtaining written informed consent from all participants.

**Table 1:** Assessments and questionnaires.

Score	Rating
Depression: Beck-Depression-Inventory II (BDI II)^23,24^	The BDI-II is scored by summing the highest ratings for each of the 21 symptoms. Each symptom is rated for the past two weeks including the present day on a four-point rating scale (0–3). Sum scores range from 0 to 63. Scores between 0 and 13 indicate minimal, between 14 and 19 mild, between 20 and 28 moderate, and between 29 and 63 severe depression.
Cognition: Montreal Cognitive Assessment (MoCA)^25,26^	The MoCA is a common cognitive screening with high sensitivity for differentiation between normal aging and mild cognitive impairment (www.mocatest.org). It is scored out of 30 points, with higher scores reflecting better performance. Different cutoffs are proposed to differentiate normal cognitive function from cognitive impairments.
Mobility: Timed up and Go Test (TUG-test)^27^	The TUG-test assesses mobility and locomotor performance. Subjects were observed and timed from the instant they rose from an armchair, walked 3 metres, and returned to a fully seated position in the chair. Subjects wore their regular footwear and were allowed to use the arms of the chair to get up.
Personality: Big Five Inventory 10 (BFI-10)^28,29^	The BFI-10 has five subscales with two bidirectional items for each of the big-five personality factors. The items are rated on a five-point Likert scale. Scale scores are then calculated as the participant’s mean response.
Autonomy support: Health Care Climate Questionnaire (HCCQ)^30,31^	The HCCQ is made up of 15 items using a Likert scale, with item 13 being coded in reverse. The HCCQ analyses patients’ perception of support for autonomy, competence, and relatedness. The score is calculated as a mean score, with higher scores indicating a higher level of autonomy support.
Health- related quality of life: Short Form Health Survey (SF-36)^32^	The SF-36 is a generic health-related quality questionnaire covering the last 4 weeks prior to testing. The SF-36 comprises eight concepts of health: physical functioning, role limitations due to physical problems, pain, general health perceptions, energy/vitality, social functioning, role limitations due to emotional problems, and mental health. Single dimension scores were calculated according to the predefined standardized scoring algorithms by following the instructions given by RAND Health Care (https://www.rand.org/health-care/surveys_tools/mos/36-item-short-form/scoring.html). Scoring was used in which items from each scale are summed and rescaled with a standard range from 0 to 100, where a score of 100 denotes the best health.
Short Form Health Survey (SF-12)^33,34^	The SF-12 is a short version of the SF-36. It encompasses 8 different domains in 12 items, including problems regarding both physical and social activity due to health, limitations in daily life due to physical or emotional problems, pain, mental health, vitality, and general health perception. Each domain is analyzed as the weighted sum of the corresponding items, with lower scores indicating less disability. According to Wirtz *et al*. (2018) a 2- and 3-factor solution can be calculated.
Adherence: Stendal Adherence to Medication Score (SAMS)^10,21^	The questionnaire comprises 18 items adding up to a cumulative adherence scale, with 0 indicating complete adherence and 72 complete non-adherence. Each item is answered on a 5-point Likert scale ranging from 0 to 4, with higher scores indicating lower adherence/higher nonadherence. Different aspects of adherence are covered, such as intentional modification of medication (items 4, 7, 8, 9, 10, 11, 12, 13, 17), lack of knowledge (items 1, 2, 3, 5) and forgetting to take the medication (items 6, 14, 15, 16, 18). The additional 5 items (19–23) that can be included in the SAMS to measure adherence to procedures in a stationary hospital context were not included in this dataset, as the focus lies strictly on medication adherence

The following variables were recorded from medical records: age, gender, main neurological diagnosis, and medication regime at admission and discharge.

The following variables were recorded via self-report in the first survey: marital status (single, divorced/widowed/living apart, and married), living condition (alone and not alone), educational level (high: German abitur or university; medium: German Realschule or general certificate of secondary education; and low: German Hauptschule or did not enter school), employment status, and number of medications per day (in the morning, noon, and evening), medical diagnoses, use of walking aids, use of visual aids, use of other aids, regular physiotherapy (yes/no), regular occupational therapy (yes/no), regular speech therapy (yes/no), frequency of consultation of neurologist (or general physician if neurologist is not available), SAMS, Beck Depression Inventory II (BDI), Big Five Inventory (BFI), Health Care Climate Questionnaire (HCCQ), and Short Form Health Survey (SF-36) (detailed in Table 1).

The following variables were recorded via face-to-face interview and assessment by trained study staff: changes of medication in the last 6 months before hospital admission (yes/no/unknown, if yes what kind of change and who did the medication change), timed-up-and-go-test, and MoCa.

The follow-up interviews were performed via telephone. Three attempts were made to reach the participants. The collected data included a semi-structured interview about medication changes from discharge (prevalence, reasons, and kind of change), selected questions from the SAMS (to address knowledge about medication, intentional modification of medication, and forgetting of medication), and SF-12.

### Ethical approval

The research protocol for this study was approved by the local ethics committee (5290-10/17). All procedures performed in the study were in accordance with the ethical standards set by the European Union under Horizon 2020 (EU General Data Protection Regulation and FAIR Data Management). Participants were advised of their voluntary participation and anonymous outcomes. Written informed consent was obtained from all participants involved in the study.

## Data Records

The dataset resulting from the study comes in an Excel spreadsheet format and is available to registered users from the ReShare data collection of the UK Data Service (https://reshare.ukdataservice.ac.uk/856032/)^11^ after permission from the research team.

Missing values are indicated with blanks. Each row represents one respondent and each column represents a variable (i.e., one column for each survey question for each phenomenon and one column for each socio-demographic variable). Detailed information on variable specifications is included in the data file and the legend document. Survey forms are stored in the English translation.

The full dataset contains potentially identifiable information regarding the participants. Therefore, the following steps were performed to avoid deanonymization:

1. Date of assessments was not reported
2. Qualitative answers from the interview were not reported.
3. While the original age is included, for ease of use, age was additionally grouped into ranges of 5 years.
4. In addition to each individual diagnosis, neurological main diagnoses were grouped into the following: cerebrovascular disorders, neuromuscular disorders, epilepsy, movement disorders, others. Rare diagnoses are not reported to avoid deanonymization
5. Timed-up-and-go test time was grouped into <20 s, 20–30 s, >30 s, and inability to perform the test due to medical reasons.
6. The use of physical, occupational, or speech therapy was combined into one variable: use of non-medical treatments (yes/no).
7. From the follow-up interviews, the following items were reported: change of medication since discharge (yes/no) and if the medication was changed, then who performed these changes (answers were classified into patient, physician, or others). No detailed information on physicians or treatments post-discharge are reported.
8. Survival of participants at follow-up was not reported as only two participants died during study period.

## Technical Validation

### Baseline characteristics of included patients

A total of 910 adults participated in the study, consisting of 389 female and 521 male patients aged 70.1 (SD 8.6) years. Most patients were married, pensioned, lived together with family members, and had a high or middle educational level (Table 2). The main neurological diagnoses derived from the patients’ medical records were movement disorders (n = 303; 33.3%), cerebrovascular disorders (n = 233; 25.6%), neuromuscular and peripheral neurological disorders (n = 168; 18.5%), epilepsy (n = 48; 5.3%), and miscellaneous diagnoses (n = 158; 17.4%) (Table 3). An overview of the SAMS items is given in Table 4.

**Table 2:** Clinical and demographical characteristics (N = 910).

Baseline data					
Metric parameters	M	SD	Range	CI 95%	Missing
Age	70.1	8.6	96.0 – 55.0	69.6; 70.7	0
Number of pills/day	5.6	3.7	20.0 - 0	5.4; 5.9	67
SAMS	6.3	7.9	72.0 - 0	5.8; 6.9	155
BDI	9.9	7.5	49.0 - 0	9.4; 10.4	1
HCCQ-D	5.6	1.1	7.0 – 0.9	5.5; 5.6	79
MoCA	23.6	2.7	30.0 – 19.0	23.4; 23.8	0
Timed Up & Go (sec)	10.5	4.3	37.0 – 5.0	10.1; 10.8	325*
**Categorical parameters**			**n**	**%**	**Missing**
Sex	female		389	42.7	0
	male		521	57.3	
Marital status	single		55	6.1	12
	widowed/divorced		222	24.7	
	married		621	69.2	
Living situation	alone		204	24.1	65
	not alone		641	75.9	
Education	high		321	35.3	14
	middle		313	34.4	
	low		262	28.8	
Occupation status	no work		756	84.0	10
	working		144	16.0	
Diagnosis group	movement disorder		303	33.3	0
	cerebrovascular disorder		233	25.6	
	epilepsy		48	5.3	
	neuromuscular		168	18.5	
	others		158	17.4	
Preparation of medication	independent		706	77.6	31
	needs help from others		141	15.5	
**Regular use of**					
walking aids	Yes		297	32.6	66
visual aids	Yes		596	65.5	67
physiotherapy	Yes		422	46.6	0
occupational therapy	Yes		134	14.7	0
speech therapy	Yes		59	6.5	0
**Follow up 1 month after discharge**			**n**	**%**	**Missing**
Medication change since discharge	Yes		204	28.3	188
	No		518	71.7	
**Follow up 12 months after discharge**			**n**	**%**	**Missing**
Medication change since discharge	Yes		322	48.5	246
	No		342	51.5	

Note: *Timed-up-and go not performed in 325 subjects for medical reasons. Stendal Adherence with medication score (SAMS), Beck Depression Inventory II (BDI), Health Care Climate Questionnaire (HCCQ), Montreal Cognitive Assessments (MoCA).

**Table 3:** Specification of neurological diagnoses.

Movement disorders	n	%
Parkinson’s disease	215	23.6
Atypical/Secondary Parkinson syndromes	45	4.9
Tremor, Dystonia, Other	43	4.7
**Cerebrovascular disorders**		
Acute neurovascular disorder (transient ischemic attack, minor stroke)	173	19.0
Chronic neurovascular disorder (arterial stenosis)	60	6.6
Epilepsy		0.0
Idiopathic epilepsy	4	0.4
Structural, secondary epilepsy	44	4.8
**Neuromuscular disorders**		
Motor neuron diseases	21	2.3
Other neuromuscular disease	24	2.6
Peripheral neuropathy	123	13.5
**Others**		
Obstructive sleep apnea (OSA)	30	3.3
Spinal disorders	18	2.0
Other	110	12.1
Total	910

**Table 4:** Stendal Adherence to Medication Score (SAMS) scores.

SAMS Items	Mean	SD	Floor %	Ceiling %	Missing
Sum Score	6.31	7.63	21.1	0.2	0
Item 1	0.45	0.95	75.1	2.5	5
Item 2	0.53	1.13	76.5	6.0	6
Item 3	0.28	0.81	84.2	2.5	9
Item 4	0.24	0.71	85.3	2.0	5
Item 5	0.51	1.07	74.5	4.1	11
Item 6	0.63	0.75	49.8	0.5	6
Item 7	0.80	1.30	62.7	10.2	83
Item 8	0.33	0.87	82.4	3.0	13
Item 9	0.30	0.80	82.2	2.2	11
Item 10	0.05	0.40	96.9	0.8	9
Item 11	0.29	0.82	84.2	2.7	8
Item 12	0.28	0.80	84.3	2.6	13
Item 13	0.19	0.66	88.1	1.6	16
Item 14	0.32	0.77	74.6	2.1	46
Item 15	0.35	0.78	70.0	1.5	84
Item 16	0.47	0.83	64.9	2.0	45
Item 17	0.17	0.64	88.8	1.8	20
Item 18	0.25	0.65	76.9	1.0	65

Note: For individual items, floor effects indicate answer levels of 0, equalling adherence, ceiling effects indicate answer levels of 4, signalling nonadherence.

### Consistency and validity of health-related QoL

The essential data concerning distribution, missing, and internal consistency of the SF-36 are given in Table 5. Internal consistency of the SF-36 subscales was evaluated using the Cronbach’s coefficient α. Internal consistency was considered adequate if Cronbach’s coefficient α values were >0.70^12^. Floor and ceiling effects were defined as the proportion of respondents scoring the highest (ceiling) or lowest (floor) possible score across any given domain. Floor and ceiling effects considered present if at least 15% of respondents reached the lowest or the highest possible score, respectively^12^.

**Table 5:** Short Form Health Survey (SF-36) Scores and internal consistency.

SF-36 subscales	Mean	SD	Floor %	Ceiling %	Missing %	Cronbach α
Physical Functioning	47.9	30.7	7.2	3.2	0.8	0.941
Social Functioning	71.0	27.3	2.2	30.7	0.3	0.803*
Role Limitations Due To Physical Problems	30.2	39.8	56.4	18.5	4.0	0.893
Role Limitations Due To Emotional Problems	61.9	45.2	31.0	55.0	3.8	0.925
Emotional Well-Being	65.1	19.2	0	1.7	1.2	0.809
Vitality	48.5	20.0	0.9	0.7	1.2	0.776
Pain	54.7	31.1	4.2	19.7	0.3	0.868*
General Health	44.4	16.7	0.2	0	1.5	0.496

Note: *Spearman Brown Coefficient. Short Form Health Survey (SF-36).

Convergent validity was measured by calculating the Spearman correlation coefficient of all SF-36 subscale scores with BDI. Results were in line with earlier studies in other cohorts^13,14^. Missing data rates were low (≤5%) for all subscales. Cronbach’s coefficients α, and were greater than 0.70 for all except subscales. Ceiling effect was present for SF-36 subscales of Social Functioning, Role Limitations Due To Physical Problems, Role Limitations Due To Emotional Problems, and Pain. Floor effect was present for SF-36 subscales Role Limitations Due To Physical Problems and Role Limitations Due To Emotional Problems. As in previous studies, SF-36 Physical component summary scores correlated stronger with SF-36 subscales pertaining to physical health relative to SF-36 subscales pertaining to emotional health (Table 6)^13^. The SF-36 Mental component summary score correlated stronger with SF-36 subscales pertaining to emotional health than with SF-36 subscales pertaining to physical health. According to previous studies, the BDI II total scores correlated strongest with the Mental component summary score and the SF-36 subscales of mental health vitality and social functioning^13,14^.

**Table 6:** Convergent validity of the Short Form Health Survey (SF-36) questionnaire.

SF-36 subscales	BDI	SF-36 PCS	SF-36 MCS
Physical Functioning	−0.331^*^	0.838^*^	0.106^*^
Social Functioning	−0.517^*^	0.348^*^	0.642^*^
Role Limitations Due To Physical Problems	−0.338^*^	0.678^*^	0.284^*^
Role Limitations Due To Emotional Problems	−0.439^*^	0.163^*^	0.771^*^
Mental Health	−0.627^*^	0.224^*^	0.812^*^
Vitality	−0.532^*^	0.477^*^	0.585^*^
Pain	−0.323^*^	0.721^*^	0.230^*^
General Health	−0.370^*^	0.545^*^	0.289^*^
Physical component summary	−0.270^*^	—	−0.026
Mental component summary	−0.591^*^	−0.026	—

Note: Spearman rank-order correlation. Beck Depression Inventory II (BDI), Short Form Health Survey (SF-36), Physical component scale (PCS), Mental component scale (MCS). *p-values <0.01.

The advantage of our study is the inclusion of people with and without cognitive deficits. Given that cognitive deficits are highly prevalent in elderly adults, our approach enhances generalizability of results. Self-reports are valid even in patients with dementia; however, a general risk is observed in obtaining less valid results on self-reported outcome measures in people with dementia. We therefore analyzed the validity of the SF-36 again with regard to cognitive state and divided the cohort into patients with MoCa of ≥26 (n = 222, 24.4%) and patients with MoCa of <26 (n = 688, 75.6%). Here, no differences were found with regard to internal consistency and convergent validity (Tables 7,8). Therefore, we conclude that the self-report of 910 participants are valid and sound.

**Table 7:** Short Form Health Survey (SF-36) scores and internal consistency in people with and without cognitive deficits.

SF-36 subscales	MoCA <26					MoCA ≥26				
	M	SD	Floor %	Ceiling %	Missing %	M	SD	Floor %	Ceiling %	Missing %
Physical Functioning	47.42	30.65	7.9	3.1	0.6	49.59	30.98	5	3.7	1.4
Social Functioning	70.61	27.43	2.5	29.7	0.3	72.29	26.80	1.4	33.3	0.5
Role Limitations Due To Physical Problems	29.88	39.98	57.0	18.9	3.9	31.22	39.37	54.5	17.4	4.1
Role Limitations Due To Emotional Problems	59.72	45.97	33.5	53.2	3.8	68.86	42.28	23.0	60.6	4.1
Emotional Well-Being	64.48	19.15	0	1.6	1.2	67.18	19.23	0.5	1.8	1.4
Vitality	48.24	20.10	1.2	0.9	1.2	49.38	19.58	0.9	0	1.4
Pain	54.03	31.16	4.6	19.0	0.3	56.91	31.01	3.2	22.2	0.5
General Health	44.23	16.11	0.1	0	1.5	44.84	18.41	0.5	0	1.8

Note: Montreal Cognitive Assessment (MoCA), Short Form Health Survey (SF-36).

**Table 8:** Convergent validity of the Short Form Health Survey (SF-36) questionnaire in people with and without cognitive deficits.

SF-36 subscales	MoCA <26			MoCA ≥26		
	BDI	SF-36 PCS	SF-36 MCS	BDI	SF-36 PCS	SF-36 MCS
Physical Functioning	−0.344^*^	0.832^*^	0.133^*^	−0.286^*^	0.852^*^	0.016
Social Functioning	−0.506^*^	0.337^*^	0.659^*^	−0.549^*^	0.385^*^	0.586^*^
Role Limitations Due To Physical Problems	−0.334^*^	0.672^*^	0.323^*^	−0.349^*^	0.695^*^	0.168^*^
Role Limitations Due To Emotional Problems	−0.436^*^	0.170^*^	0.771^*^	−0.449^*^	0.138^*^	0.769^*^
Mental Health	−0.609^*^	0.241^*^	0.805^*^	−0.685^*^	0.162^*^	0.825^*^
Vitality	−0.497^*^	0.506^*^	0.553^*^	−0.636^*^	0.384^*^	0.680^*^
Pain	−0.313^*^	0.731^*^	0.234^*^	−0.354^*^	0.694^*^	0.195^*^
General Health	−0.349^*^	0.548^*^	0.256^*^	−0.437^*^	0.540^*^	0.380^*^
Physical component summary	−0.266^*^	1.000	−0.006	−0.279^*^	1.000	−0.090
Mental component summary	−0.580^*^	−0.006	1.000	−0.628^*^	−0.090	1.000

Note: Spearman rank-order correlation. Montreal Cognitive Assessment (MoCA), Beck Depression Inventory II (BDI), Short Form Health Survey (SF-36), Physical component scale (PCS), Mental component scale (MCS). *p-values <0.01.

### Measurement of adherence (SAMS)

It is important to mention that adherence was measured using a single-source approach with a self-report questionnaire, as the key focus of the present dataset lies on understanding patient-related barriers and difficulties concerning medication adherence that is not possible to understand with administrative data or objective adherence measures. Due to its subjectiveness and complexity, no gold standard for measuring adherence is agreed upon^15^, but research shows that self-reports are comparable to objective measures and can provide valid information on adherence that is clinically useful, especially when the items are derived from a strong theoretical model and are validated, as is the case with the SAMS^16–18^. Both self-reports and electronic monitoring have been shown to over- and underestimate adherence^18^,^19^. Additionally, objective reports such as electronic pill counts are not always feasible, especially in daily clinical practise or older adults in an inpatient setting^19,20^. Additionally our dataset provides information on other measures such as quality of life, depression, cognition and relevant sociodemographic information, which are all strongly linked to adherence^7^.

In addition to providing the responses to each SAMS item, the current dataset also presents SAMS sum scores. Sum scores are left blank in case of missings in one of the SAMS items. However, it is important to point out that while omitting these sum scores does not affect the overall mean SAMS score, it leads to an even lower number of patients with higher SAMS scores. Therefore, we encourage each researcher to make an educated decision on whether or not they want to include sum scores despite missing values, depending on their respective research question and the data needed to approach it. The SAMS manual^21^ describes several possibilities for calculating adherence, suggesting that only a total score of 0 defines adherence, whereas higher values indicate different degrees of nonadherence. Thus, a deviation of 1 or 2 points due to missings is unlikely to change an individual’s overall classification into adherent vs. nonadherent according to the SAMS. Additionally, it is possible to calculate subscores of adherence elucidating the roles of forgetting, modification and missing knowledge of medication^21,22^. Likewise, missing items should not alter the classification of patients into these subgroups, and by providing information on all items we encourage researchers to utilize the dataset in a way that best suits their interests.

## Usage Notes

This dataset provides a plethora of opportunities to explore multiple facets related to adherence, social, or psychological aspects of hospitalized older adults. It also provides insights into patient´s concerns during the transition from inpatient to outpatient care.

Following are a non-exhaustive list of scientific questions that can be addressed with the help of this dataset:

- What are the patterns of depression and cognitive ability in hospitalized older adults with neurological disorders? What is their relationship with other health-related and psychosocial measures?
- What determines patient-physician relationship using the HCCQ?
- What is the relationship between adherence and health-related QoL? At which adherence thresholds can an effect of nonadherence on health-related QoL observed?

## Data Availability

The data directly describe the patients’ answers given in a numerical format and no analyses were performed, therefore no custom code was necessary to generate or process the data.
